# Global transcriptome analysis of H5N1 influenza virus-infected human cells

**DOI:** 10.1186/s41065-019-0085-9

**Published:** 2019-02-06

**Authors:** Ying Cao, Kun Zhang, Lirong Liu, Wei Li, Bin Zhu, Shuang Zhang, Ping Xu, Wenjun Liu, Jing Li

**Affiliations:** 1 School of Life Sciences, University of Science and Technology of China, Hefei, China; 2 CAS Key Laboratory of Pathogenic Microbiology and Immunology, Institute of Microbiology, Chinese Academy of Sciences, Beijing, China; 3 Philips Institute for Oral Health Research, School of Dentistry, Virginia Commonwealth University, Richmond, Virginia USA; 4 University of Chinese Academy of Sciences, Beijing, China

**Keywords:** H5N1 influenza virus, Differentially expressed genes, Signaling pathway, Transcriptomic

## Abstract

**Background:**

Influenza A virus (IAV) belongs to the *Orthomyxoviridae* family. IAV causes a highly contagious respiratory disease in humans that exacts severe economic losses globally. The virus uses strategies developed to exploit and subvert cellular proteins and pathways to increase its own replication and to inhibit antiviral immune response.

**Results:**

A/bar-headed goose/Qinghai/1/2005 (A/QH) was able to infect A549 and 293 T cells, with a high infection rate for A549 cells. To identify host cellular responses of human cells to influenza infection, differentially expressed genes (DEGs) between AIV-infected groups and uninfected controls were identified using RNA-sequencing. The DEGs were annotated by Gene Ontology and the Kyoto Encyclopedia of Genes and Genomes pathway analyses, which revealed that the DEGs were mainly linked to cellular function and metabolic processes, while the cellular function that is probably associated with host cellular response of human cells, including defense response to virus and protein modification. All the DEGs and pathways were possibly involved in the response to IAV invasion.

**Conclusions:**

The global transcriptome analysis results revealed that sensitive genes and pathways of the cells were infected with the influenza virus and provided further evidence to investigate the complicated relationship between IAV and host cells.

**Electronic supplementary material:**

The online version of this article (10.1186/s41065-019-0085-9) contains supplementary material, which is available to authorized users.

## Background

Influenza is one of the commonest respiratory infectious diseases of humans. Influenza A virus (IAV) is a negative-sense, single-stranded, enveloped RNA virus that belongs to the influenza A genus of the *Orthomyxoviridae* family. The threat of IAV to human health is significant and continual, which could be in the form of seasonal epidemics and occasional pandemics [1]. It has been estimated that more than 50 million people globally have died from influenza [2]. The highly pathogenic avian influenza (HPAI) H5N1 has infected thousands of people and spread globally in transported poultry or via indirect contact [3, 4]. Currently, it is impossible to eliminate the virus during its infection [5].

Host defenses against IAV infection are provoked by the innate immune system after virus attachment to target cells [6]. During infections of viruses, innate immune responses are triggered when viruses or their genetic material are detected by cellular pattern recognition receptors of the innate immune system [7–9]. Influenza viruses such as H5N1 and the pandemic 2009 H1N1 influenza virus could infect and induce excessive inflammatory immune responses which are associated with elevated morbidity as well as mortality [10, 11]. However, the underlying molecular mechanisms of the regulation of the balance between the protective and pathological immune responses under infection with various strains of influenza virus are still required to be elaborated.

Transcriptomic analyses of the host cellular responses to viruses infection can be employed to investigate potential cellular factors that are related to viral infection either directly or indirectly [12]. RNA-sequencing (RNA-seq) technology, a recently developed transcriptome profiling approach, combined with bioinformatics has become an important tool in the exploration of cellular signaling mechanisms. RNA-seq technology can be used to uncover dynamic alterations in the pathogen genome itself and systemic changes in host gene expression profiles during the process of infection by pathogens. Thus, RNA-seq technology could be useful in identifying the pathogenesis and mechanisms of the infection and interaction of pathogens. RNA-seq has been applied to study various viral infections and diseases, and previous studies primarily focused on the differing virulence of influenza virus strains [13, 14].

In the current study, RNA-seq was employed to annotate host responses to infection with H5N1 influenza viruses in 293 T and A549 cells. The differential virus replication in A549 and 293 T cells uncovered potential candidate gene that may be correlated with the observed resilience of human cells to highly pathogenic influenza virus infection. The global survey of virus type-specific and cell-specific mRNA profiles demonstrate the role and mechanism of host-virus cellular responses during influenza virus infection and demonstrate a signature response of the cells infected with the H5N1 influenza virus. We believe that this information will aid in the development of vaccines and other control strategies.

## Methods

### Virus preparation

The highly pathogenic avian influenza A virus, A/bar-headed goose/Qinghai/1/2005 (H5N1; A/QH), was stored at the Wuhan Institute of Virology, Chinese Academy of Sciences. The virus was grown in allantoic cavities of the 10-day-old, specific pathogen-free embryonated chicken eggs at 37 °C for 72 h. Virus titers in allantoic fluid were depended on calculating the 50% tissue culture infective dose (TCID_50_). Virus stocks were stored at − 80 °C.

### Cell cultures and antibodies

Human lung carcinoma epithelial cells (A549) and human embryonic kidney cells (293 T) were obtained from the China Infrastructure of Cell Line Resources. The cell lines were grown in Dulbecco’s modified Eagle’s medium (DMEM; Gibco) supplemented with 10% fetal bovine serum in 5% CO_2_ at 37 °C. A rabbit polyclonal antibody against nucleoprotein was obtained by immunizing the animals with hexahistidine-tagged nucleoprotein and matrix protein as described previously [15].

### Virus infection and the RNA preparation

For virus infection, 293 T or A549 cells were incubated with A/QH virus at a multiplicity of infection (MOI) of 0.01 at 37 °C for 1 h. The cells were washed by phosphate-buffered saline (PBS) and incubated in DMEM for the indicated times in 5% CO_2_ at 37 °C. The infected cells were collected, and RNA was extracted. The total RNA was isolated with TRIzol reagent (Invitrogen) and subjected to RNA deep sequencing using standard Illumina protocols. The RNA quality was determined by analyzing ribosomal RNA band integrity.

### RNA-seq analysis

For cDNA library preparation, the total RNA from the two cell lines was treated with RNase-free DNase I (TaKaRa Bio) following the instruction of manufacturer’s. RNA was quantified using a NanoDrop ND1000 spectrophotometer (Thermo-Fisher Scientific) and the quality was assessed using a model 2100 Bioanalyzer (Agilent). The RNA integrity number value of each sample was > 8. The two viral cDNA libraries were prepared according to the standard Illumina protocol (NEBNext® Ultra™ II RNA Library Prep Kit for Illumina®). In brief, polyA-enriched mRNA was isolated with magnetic oligo (dT) beads from 2 μg of total RNA for each sample and fragmented using divalent cations at an elevated temperature. The truncated RNAs were subjected to reverse-transcription for the first strand cDNA with random primers (Invitrogen Inc.), which was followed by second-strand cDNA synthesis. After the end repair and 3′-adenylation, a single ‘A’ base was added to the 3′ end of each cDNA fragment. This treatment was convenient for subsequent adapter-ligation with the pair–end adapters (Illumina). Following ligation, the cDNA fragments were purified by 2% agarose gel electrophoresis and enriched by 15 cycles of PCR to create the final cDNA libraries. The libraries were quantified using a DNA-1000 kit bioanalyzer (Agilent).

### Transcriptome assembly

To annotate and value the transcript abundances for the sequenced reads, the human reference genome and annotation for protein-coding genes were downloaded from the website of the University of California at Santa Cruz (http://genome.ucsc.edu) as the reference. After filtering reads containing sequencing adapters and reads of low quality, the remaining reads were aligned to the human genome using Tophat v2.0.9. The distributions of reads for known genes were analyzed using the HTSeq.

### Transcriptional profiling analysis

To analyze gene levels of 293 T or A549 cells infected with A/QH virus, the Cuffdiff (v2.1.1) program was used to quantify the fragments per kilobase of gene model per million mapped reads (FPKM) to code genes in each cell. Ingenuity Pathway Analysis (IPA) software (Ingenuity Systems) was used for differential gene expression analysis, and the FDR-corrected *p*-value < 0.05 was considered DE genes.

### GO enrichment and KEGG pathway analysis

Gene Ontology (GO) functional classifications were defined by the Blast2GO software, the enriched gene functional categories were further classified by the GO analysis, with *p-value* < 0.05. Kyoto The Encyclopedia of Genes and Genomes (KEGG) pathway database was accessed using the KOBAS software via a hypergeometric test, with a corrected *p-value* < 0.05. Q-value is used as statistical method for estimating false discovery rate (FDR), which is a conventional significance measure in the analysis of genome-wide expression data, with a corrected Q-value < 0.05.

### Immunofluorescence assays

Cells grown in a 24 well culture plate were infected with A/QH at a multiplicity of infection (MOI) of 0.01. The cells were collected at the indicated times and then fixed in 4% formalin buffer at 4 °C, followed by permeabilization with PBS containing 0.5% Triton X-100 (PBST) at room temperature for 10 min. The slides were incubated in 0.4% bovine serum albumin at 37 °C for 1 h, and then incubated with anti-NP antibody at 37 °C for 1 h. After washing with PBST for 1 h, the cells were incubated with fluorescein isothiocyanate-labeled goat anti-rabbit antibody diluted in PBST at 37 °C for 1 h, followed by washing with PBST for 1 h. Cell nuclei were stained with 4′,6-diamidino-2-phenylindole (DAPI) for 20 min and observed using a model LSCMFV500 confocal laser scanning fluorescence microscope (Olympus).

### Host gene expression analysis by quantitative PCR

Total RNA was extracted using TRIzol reagent (Invitrogen). RNA was reverse transcribed to cDNA using the transcriptase kit (Transgen) and subjected to quantitative PCR using SYBR Green PCR master mix (TOYOBO). The relative expression of mRNA was normalized by β-actin. The primers for eight target genes (Table 1) were designed by using Primer Premier 5.

**Table 1:** Primer sequences used for qPCR

Gene Name	Forward Primer (5′- 3′)	Reverse Primer (5′- 3′)
RIN1	GCACCTGGCGAGAGAAAAG	TAGATTTCCGCACGAGGAACG
TNF-α	GGAGAAGGGTGACCGACTCA	TGCCCAGACTCGGCAAAG
IFIT2	AAGCACCTCAAAGGGCAAAAC	TCGGCCCATGTGATAGTAGAC
ELOVL3	CTGTTCCAGCCCTATAACTTCG	GAATGAGGTTGCCCAATACTCC
ISG15	CGCAGATCACCCAGAAGATCG	TTCGTCGCATTTGTCCACCA
IFIT1	TTGATGACGATGAAATGCCTGA	CAGGTCACCAGACTCCTCAC
AKR1C3	GTCATCCGTATTTCAACCGGAG	CCACCCATCGTTTGTCTCGTT
TXN	GTGAAGCAGATCGAGAGCAAG	CGTGGCTGAGAAGTCAACTACTA

### Plaque assay

MDCK cells were seeded in 12-well plates and infected with serial dilutions of the virus in serum-free DMEM supplemented with 4 μg/mL of TPCK-trypsin for 2 h, and then washed with PBS. The cells were covered with Modified Eagle’s Medium containing 1% agarose (AMRESCO) and 2 μg/mL of TPCK-trypsin. The plates were allowed to solidify at 4 °C for 5 min and incubated upside-down at 37 °C. After 72 h, viral titers were determined through counting the visible plaques.

## Results

### Differential viral replication in human cells

**Fig. 1:**
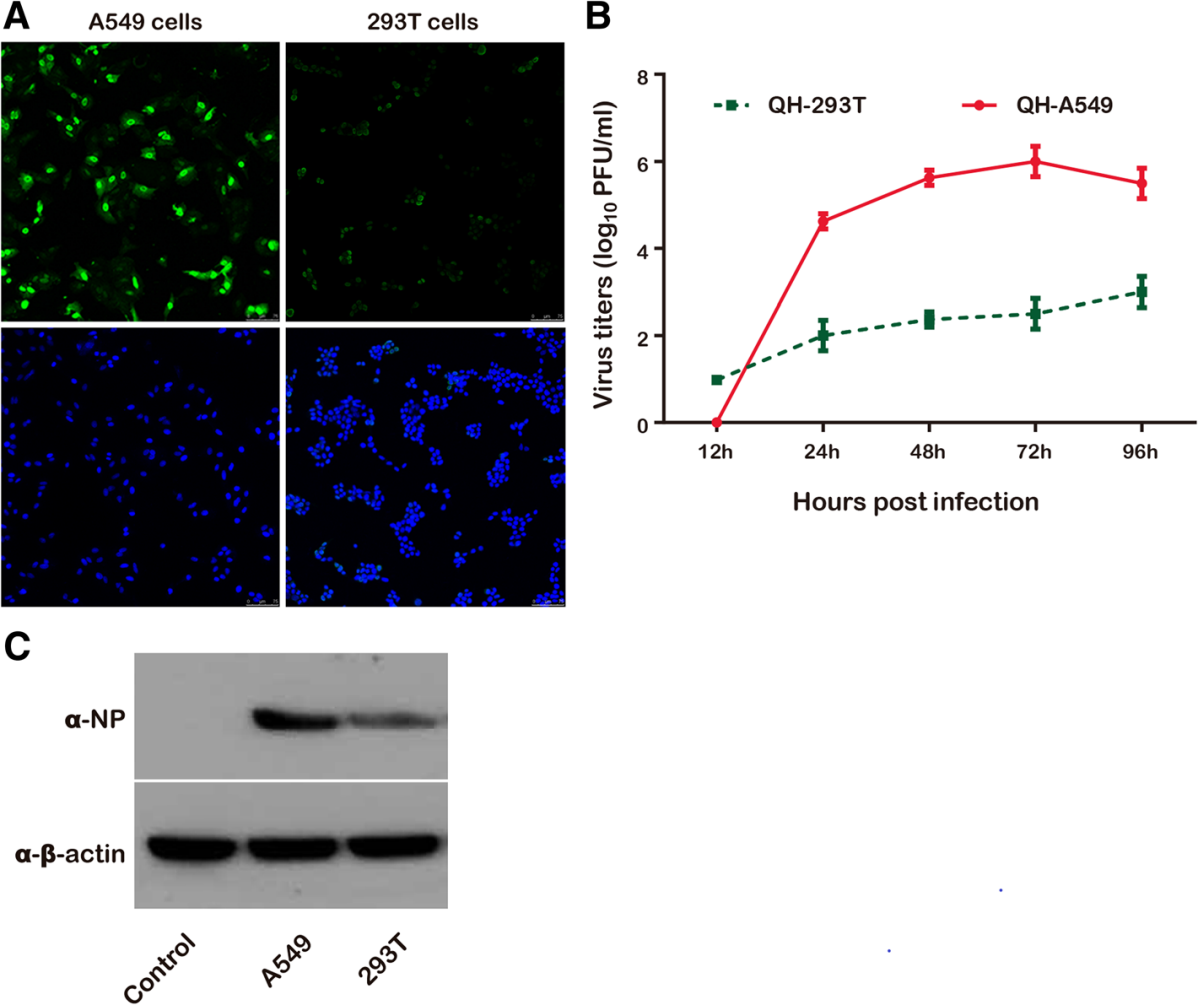
Differential H5N1 virus replication in A549 or 293 T cells. **a** Immunofluorescence staining of A549 and 293 T cells at 36 h post-infection with A/QH at a MOI of 0.01. The influenza virus NP protein was analyzed with FITC-conjugated antibody.(top). The Nuclei was examined using DAPI staining (bottom). **b** Growth curve of influenza A viruses in A549 and 293 T cells. The cells were infected with A/QH virus (MOI of 0.01). Culture supernatants were collected at the indicated times and viral titers were determined by plaque forming units. **c** The cells were collected at 18 h after infection. The Western blot analysis was used to determine NP protein levels. β-actin is used as a control

By inoculating A549 and 293 T cells with A/QH virus at a MOI of 0.01, the virus replication in human cells was determined. The ratio of infected cells was identified by measuring viral NP using immunofluorescence microscopy analysis. The results indicated that the virus titer of A549 cells was obviously higher than that of cells infected in 293 T cells (Fig. 1a). To confirm viral replication, A549 and 293 T cells were inoculated with A/QH viruses, respectively, followed by the examination of their replication kinetics. Aliquots of the cell supernatant were harvested at 12, 24, 48, 72, and 96 h post-infection. A plaque assay was used to examine viral titers. A/QH virus replicated poorly in 293 T cells (Fig. 1b), with virus titers more than 1000-fold lower than the titers in A549 cells, indicating less efficient H5N1 viral replication in 293 T cells. These results were further confirmed by the analysis of NP protein expression levels by Western blots; and, it was found that A/QH virus infecting A549 cells displayed higher NP expression, which is consistent with the staining analysis, (Fig. 1c).

### RNA-seq analysis and reads mapping

We extracted total RNA, and built up sequencing libraries to perform deep sequencing. The Illumina-based RNA-seq was performed on the Hiseq4000 platform, and three sets (repeated 3 times for each group) of different cell samples were used. Three biological replicates were performed in each set. The sequence obtained by sequencing was used for subsequent analysis. Finally, the unique gene location or multiple genome locations could match total cell samples and control reads (Table 2).

**Table 2:** Summary of RNA-Seq data

Sample	Raw reads	Clean reads	Clean bases	Error rate (%)	Q20 (%)	Q30 (%)	GC content (%)
A549-8 h	29,385,754	28,948,578	4.12G	0.02	96.89	89.67	52.65
A549-8 h	30,875,826	29,048,576	4.34G	0.02	95.48	89.21	52.10
A549-8 h	28,045,738	27,746,588	4.31G	0.01	97.46	91.73	52.17
A549-0 h	29,947,344	28,298,487	4.15G	0.02	97.29	90.50	52.47
A549-0 h	29,678,857	28,875,243	4.35G	0.01	96.87	89.87	52.12
A549-0 h	28,985,085	27,856,326	4.52G	0.01	96.86	91.78	52.32
293 T-8 h	27,094,852	26,827,590	3.97G	0.01	95.38	88.84	52.55
293 T-8 h	29,947,576	27,948,592	3.68 g	0.02	96.49	89.17	52.71
293 T-8 h	26,048,570	25,958,796	3.86G	0.01	95.82	89.84	52.62
293 T-0 h	27,406,945	26,396,898	3.95G	0.01	95.43	89.14	52.48
293 T-0 h	29,094,048	28,194,512	3.72G	0.01	95.50	89.68	52.24
293 T-0 h	28,395,329	26,998,396	3.84G	0.02	96.93	88.98	52.61

### Global changes in expression in response to host genes

**Fig. 2:**
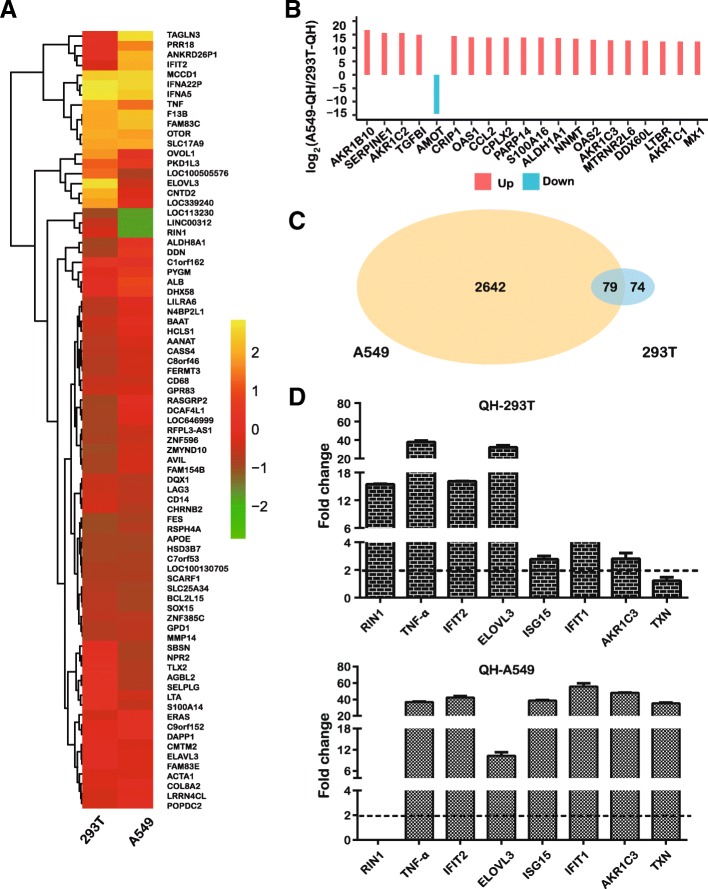
Identification and characterization of IAV infection. **a** A549 or 293 T cells were infected with A/QH viruses at an MOI of 0.01 for 8 h. Total RNA was extracted and used for RNA-seq analysis. The expression values shown in shades of yellow and green indicate gene level above and below the median expression value across all the samples (log scale 2, from − 2 to + 2), respectively. **b** The 20 highest fold-changes of the RPKM value of gene expression in A549-QH and 293 T-QH was sorted (log scale 2, from A549-QH to 293 T-QH). **c** qRT-PCR analysis of the expression of selected genes in A/QH virus-infected A549 or 293 T cells relative to uninfected controls. The fold-difference was measured by the 2^-△△Ct^ method; RNA levels were normalized to corresponding β-actin. Error bars represent standard deviation. **d** Venn diagram showing the distribution of shared differentially expressed genes in 293 T and A549 cells infected with A/QH virus

RNA isolates were individually prepared from both H5N1 virus and mock infected A549 or 293 T cells. To identify their functions during viral infection, RNA deep sequencing was performed to analyze the total RNA profiles in uninfected or H5N1 infected A549 and 293 T cells. As shown in Fig. 2a, a total of 1299 promoted and 1422 suppressed differentially expressed genes (DEGs) were identified in A549 cells infected with A/QH virus. In contrast, 293 T cells showed relatively few DEGs, with 144 promoted and 9 suppressed genes (Additional file 1: Table S1).

To compare gene expression profiles between A549 and 293 T cells, we filtered out genes with low expression (FPKM = 0.001), calculated the fold-changes in RPKM value of each gene from the two samples, we used the statistical enriched and listed the top 20 fold-changes by the absolute value of log scale 2 obtained from both cell lines (Fig. 2b). The Venn diagram shown in Fig. 2c illustrates the overlap of A549 and 293 T cells responding to A/QH virus infection induced DEGs, which may help to investigate the various influences induced by virus infection. Up-regulated genes in A529 and 293 T cells included 2721 genes and 153 genes, respectively, with 79 genes common to both cell types. These results indicated a difference in human cells upon virus infection, with some shared alterations in expression among different cell types.

### Confirmation of DEGs by quantitative RT-PCR

By performing quantitative RT-PCR (qRT-PCR) on selected DEGs, the differential expression profiles of genes obtained by RNA-seq analysis were validated. Although minor differences were observed between these two types of analysis due to their intrinsic differences, the results of these two analyses demonstrated the same relative regulation of DEGs. Consistent with virus propagation over time, most of the DEGs displayed a higher level of mRNA in A549 cells than that in 293 T cells (Fig. 2d).

pro-inflammatory cytokines and chemokines production induced by A/QH infections have been considered to be linked to elevated morbidity and mortality in humans. As shown in Fig. 2d, the expression of tumor necrosis factor-alpha was not changed in infected A549 or 293 T cells. However, the levels of AKR1C3, TXN, and the interferon response-related genes IFIT2, IFIT1, as well as ISG15 were markedly elevated in A/QH-infected A549 cells in comparison with infected 293 T cells. The expression of RIN1 and ELOVL3 in 293 T cells was higher than in A549 cells. These findings suggested a remarkable initiation of the response of the host gene in A/QH-infected A549 cells.

### GO and KEGG pathway enrichment analyses based on DEGs

By importing datasets representing genes with changed expression profiles obtained from RNA-seq analyses for the analyses of GO and KEGG pathway enrichment, we examined possible biological interactions of DEGs and determined important functional networks by A/QH infection in human cells. The results of GO analysis of the five most common the subclasses of cellular processes are presented in Tables 3 and 4. The most common cellular process was biological regulation in infected A549 cells and chronic inflammatory response to antigenic stimulus in infected 293 T cells. The cellular process, which was in the biological process category, was most significantly regulated by A/QH infection.

**Table 3:** Gene ontology (GO) enrichment for differentially expressed genes (DEGs) upon A/QH virus infection in 293 T

Top	GO NO.	*P* Value	log (*P* Value)	Numbers of DEGs
1	GO:0002439 (chronic inflammatory response to antigenic stimulus)	0.05127	2.970649493	2
2	GO:0006547 (histidine metabolic process)	0.2533	1.373180722	2
3	GO:0009075 (histidine family amino acid metabolic process)	0.3532	1.04072081	2
4	GO:0002544 (chronic inflammatory response)	0.46905	0.757045906	2
5	GO:0016337 (cell-cell adhesion)	0.59023	0.527242988	7

**Table 4:** Gene ontology (GO) enrichment for differentially expressed genes (DEGs) upon A/QH virus infection in A549

Top	GO NO.	*P* Value	log (*P* Value)	Numbers of DEGs
1	GO:0065007 (biological regulation)	5.16E-19	42.10818	1082
2	GO:0034097 (response to cytokine stimulus)	7.24E-19	41.7695	81
3	GO:0002376 (immune system process)	3.64E-18	40.15455	261
4	GO:0050789 (regulation of biological process)	8.15E-18	39.34851	994
5	GO:0050896 (response to stimulus)	1.76E-17	38.57863	885

**Fig. 3:**
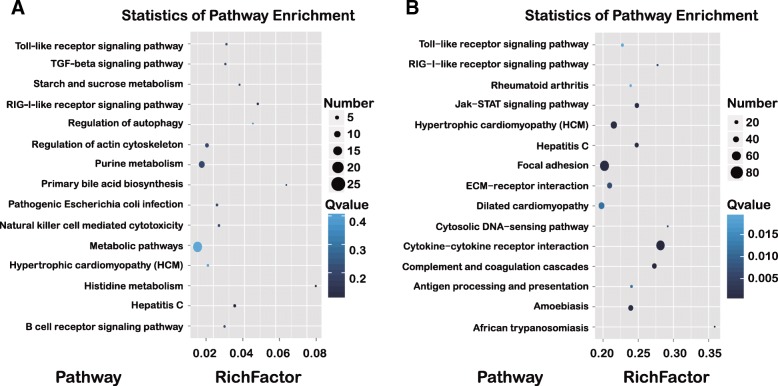
Top 15 enriched pathways based on DEGs in A/QH-infected cells. Pathway analysis allowed the construction of a scatter plot of KEGG pathway enrichment statistics for DEGs following A/QH infection of 293 T cells (**a**) and A549 cells (**b**). Rich factor is the ratio of the number of differentially expressed genes noted in the pathway terms to all gene numbers noted in this pathway term. A greater Rich Factor indicates higher intensiveness. Q-value is the corrected *p*-value ranging from 0 to 1 (blue). A lower Q-value indicates higher intensiveness

Furthermore, DEGs were mapped into the KEGG pathway database to further explain the individual function analysis. Hundreds of signaling pathways was found to be enriched in cells, among which 32 pathways in 293 T cells and 60 pathways in A549 cells were found to be significantly altered (*Q*-value< 0.05 and *p* < 0.01). The top 20 enriched pathways in A/QH-infected A549 or 293 T cells are summarized in Fig. 3. Metabolic pathways and purine metabolism signaling pathway are high associated with the responses to A/QH infection in 293 T cells (Fig. 3a). In A549 cells, many receptor-interaction signaling pathways were enriched, including the cytokine-cytokine receptor interaction, the Toll-like receptor-downstream pathway, and the Jak-STAT signaling cascade (Fig. 3b).

**Fig. 4:**
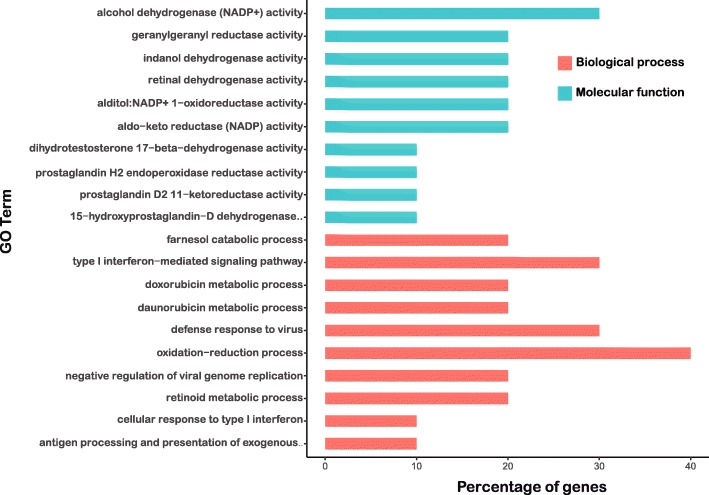
GO functional enrichment of unique differentially expressed genes in A549 cells. GO enrichment of unique differential expression genes indicated that biological processes and molecular function were most enriched, judging by their *p*-values

GO analyses have also been employed to reveal the possible functions of the identified unique gene transcripts in cell samples. With the examinations of the differential unique genes in the A/QH group relative to cellular function and metabolic processes in A549 cells, we found that the most enriched biological processes mainly include the oxidation-reduction process, defensive response to viral invasion, and the signaling pathway mediated by type I interferon (Fig. 4), with alcohol dehydrogenase (NADP+) activity and dehydrogenase activity as the most significant molecular functions (Additional file 2: Table S2 and Additional file 3: Table S3).

## Discussion

The highly pathogenic avian IAV strain, H5N1, can cause severe disease and death, especially in older individuals in poor health with other underlying diseases [16]. Host barriers have hindered the efficient growth of influenza virus from one species in other species [17]. The receptor specificity of hemagglutinin and the internal genes of influenza virus are critical in host-range restriction. In addition to the virus itself, the host restriction protein has an irreplaceable role during virus replication [18, 19]. Airway epithelial cells are major target cells for H5N1 virus infection. Human A549 cells (wildly used cell lines as pulmonary epithelial cells) serving as virus-susceptible cells and 293 T cells (used as human kidney cells) serving as virus-insusceptible cells were used in the present study. H5N1 A/QH virus was used to infect A549 and 293 T cells. The virus is characterized to spread rapidly via migratory birds and is likely the major agents for several human infections throughout Europe and China in the past few years [20, 21]. Interestingly, virus propagation was higher in A549 cells than in 293 T cells. The reduced viral replication of virus in 293 T cells provided evidence to further reveal potential host factors and immunological pathways that are involved in the resistance against the infection of influenza virus, especially the H5N1 influenza virus.

Previous studies have already extensively studied the influences of influenza virus proteins on immune escape. However, the exact mechanism underlying the regulation of the balance of host responses to virus infection has not been fully elucidated [22–24]. The utilization of genome-wide profiling techniques (e.g., microarray analysis) has identified novel host factors involved in IAV infection [25]. Herein, transcriptome analysis using the RNA-seq technology was applied to identify DEGs of two host cell types during infection. Numerous genes displayed significantly altered expression levels and some common DEGs were revealed in the H5N1 virus-infected human cell lines. These results have discovered and verified a series of cellular targets that may be associated with the cellular responses to the infection of influenza virus.

Previous study suggest that the transcriptional activity of interferon-stimulated genes (ISGs) are regulated by the interferon-mediated immune response, which collectively play a significant role in innate antiviral defense [26]. The IFIT family of genes is a type of ISG, with important antiviral and immune regulation roles. We detected the up-regulation of interferon inducible proteins, including IFIT1, IFIT2, ISG15, and tumor necrosis factor-alpha (TNF-α), which are coordinately expressed in A549 cells in response to H5N1 virus. The expression levels of these ISGs showed no significant differences in 293 T cells infected with H5N1 virus. ISGs can target almost any step in influenza virus life cycle [27, 28]. Some of the most potent antiviral effectors reinforce the system by further stimulation of ISGs, inducing a “cytokine storm” in these cells due to continuous secretion of cytokines [29]. Our results demonstrate that IAV infection can cause strong innate responses in human lung cell lines sensitive to IAV infection and reveal a series of ISGs that possess relationship with IAV infection. The attenuated induction of the interferon-mediated immune mediators may limit H5N1-induced virus propagation in 293 T cells.

Metabolism, as a biological process, contribute to a series of chemical reactions that modify a specific molecule for storage. It has been shown that host cellular metabolic networks are changed by viral infections [30]. Our study demonstrate that metabolic pathways were prominent in the response of A549 cells, suggesting that some genes involved in metabolic pathways, such as AKR1C3 and TXN, play key regulatory roles. The regulatory function of the AKR1C3 gene in influenza virus infection remains to be further studied. A previous report indicated that AKR1C isoenzymes 2 and 3 may be related to the progression of hepatitis C virus-related liver disease in males. AKR1C gene expression has been implicated in the tumorigenesis of other malignancies via the regulation of metabolism [31, 32]. ELOVL3 is involved in metabolic regulation in humans [33]. The present data of pathways in 293 T cells demonstrated the significant enrichment of metabolic processes. Whether these regulations are related to the low replication capacity of the virus in 293 T cells will be our focus in future studies.

A recent clinical study revealed a significant connection between early cytokine responses, immune cell recruitment, and inferior prognoses during H5N1 infection [34]. Extensive transcriptome studies have shown that cytokine storm and uncontrolled inflammatory responses are common features of influenza virus lethality [35]. The present findings are consistent with the previous data. Reducing excessive host response appears to be a rational strategy of treating severe influenza, and drugs aimed to perform this strategy have made some progress in treating influenza. A deeper exploration of the transcriptome data of influenza virus will certainly help us discover more specific therapeutic targets or pathways leading to host overproduction of pathological transcriptional responses to influenza virus and then develop therapeutic drugs and strategies to combat lethal influenza infections.

## Conclusion

Our results highlight a reliable approach to investigate the interactions of influenza virus with the host cells. The transcriptome analysis results demonstrated sensitive genes and pathways of two human cell types infected with H5N1 influenza virus. We believe that this data will inform the development of therapeutic drugs and strategies to lessen lethal influenza infections.

## Additional files

Additional file 1: **Table S1.** Differentially expressed genes of A549 and 293T cells. (XLS 26 kb)

Additional file 2: **Table S2.** The differential unique genes in the A/QH group relative to biological process. (XLSX 46 kb)

Additional file 3: **Table S3.** The differential unique genes in the A/QH group relative to molecular function. (XLSX 37 kb)

## Abbreviations

293 T: human embryonic kidney 293 T cells
A549: human lung carcinoma epithelial cells
DEGs: differentially expressed genes
GO: Gene ontology
IPA: Ingenuity pathway analysis
KEGG: Kyoto Encyclopedia of Genes and Genomes
